# Gout and sexual function: patient perspective of how gout affects personal relationships and intimacy

**DOI:** 10.1186/s41927-019-0056-9

**Published:** 2019-02-28

**Authors:** Jasvinder A. Singh

**Affiliations:** 1 Medicine Service, Birmingham VA Medical Center, 700 19th St S, Birmingham, AL 35233 USA; 2 Department of Medicine at School of Medicine, and Division of Epidemiology at School of Public Health, University of Alabama at Birmingham, Faculty Office Tower 805B, 510 20th Street S, Birmingham, AL 35294-0022 USA

**Keywords:** Gout, Sexual function, Intimacy, Nominal groups, Qualitative, Personal relationships, Patient perspective

## Abstract

**Background:**

In absence of previous studies, we assessed how gout impacts relationship and intimacy with spouse/significant other.

**Methods:**

We enrolled a convenience sample of consecutive patients with doctor-diagnosed gout from a community-based outpatient clinic. Nominal groups were conducted until saturation was achieved. Responses were collected verbatim, discussed and then rank-ordered by each participant with votes.

**Results:**

Forty-four patients with gout participated in 14 nominal groups, seven male only groups, six female only groups and one group had people with both sexes. Overall, the mean age was 61.7 years (SD, 12.2), mean gout duration was 11.8 years (SD, 11.8), 50% were men, 68% African-American, 43% retired, 48% currently married, 94% were using either allopurinol and/or febuxostat, and 39% had had no gout flares in the last 6 months. The top five responses accounted for 75% of all votes and included physical (28%) or emotional impact (17.4%) on intimacy, disability (12.9%), issues with trust/understanding (10.6%) and social life interference (6.8%). When examining the top-rated concern for each nominal group, physical impact on intimacy was ranked top by eight nominal groups; and emotional impact on intimacy, physical function limitation, trust issues/understanding by two nominal groups each. There were no differences evident by patient gender in the concern that was top-ranked.

**Conclusions:**

Gout significantly impacts relationship and intimacy with spouse/significant other. Our observation of the physical and emotional impact of gout on intimacy should lead to studies to understand this further and assess if more optimal gout control can improve sex lives of people with gout.

**Electronic supplementary material:**

The online version of this article (10.1186/s41927-019-0056-9) contains supplementary material, which is available to authorized users.

## Background

Gout is the most common inflammatory arthritis in adults with an increasing prevalence in the US and worldwide [1, 2]. Gout leads to a significant morbidity burden and is associated with deficits in quality of life (QOL) [3–6]. In a qualitative study assessing the QOL, 40% of the nominal groups reported that gout flares negatively affected sexual function, leading to problems in having sex as well as to low or no sexual desire [7]. Recent observational studies showed that gout was associated with a higher risk of both organic and psychogenic erectile dysfunction in men [8–10]; data from women are limited. This indicated that gout may be associated with sexual dysfunction.

Sex is an important contributor to QOL [11]. Understanding patient perspective related to sexual function is important, since sexual dysfunction is often under-detected and undertreated because of barriers to the discussion about sex in doctor-patient communication and the lack of medical training in human sexuality [11]. Previous studies have reported that patients with chronic diseases have a higher risk of sexual dysfunction, related to illness, treatment and concomitant depression [11–13]. Theoretical models have been proposed for sexual dysfunction in chronic diseases to help understand the underlying constructs and mechanisms [14, 15]. The autoimmune counterpart of gout, rheumatoid arthritis is associated with a high prevalence of sexual problems [16]. Despite the evidence of sexual dysfunction in other inflammatory arthritis [17–20] and these conceptual models for chronic diseases, there is paucity of data related to sexual function in gout. We know little or nothing about how gout affects sexual life and relationships.

Our study aim was to address this important knowledge gap by performing formative work in people with gout. The study objective was to assess the effect of gout on the relationship with spouse or significant other, including the effect on intimacy, using the Nominal Group Technique (NGT). A secondary objective was to assess whether these effects differed by patient gender.

## Methods

### Study sample

The study team invited a convenience sample of consecutive patients with doctor-diagnosed gout identified with at least one outpatient visit for gout between January 2016 to August 2017 at a community-based outpatient clinic affiliated with University of Alabama at Birmingham (UAB), Birmingham, Alabama, USA. Potential study participants were identified by the presence of an International Classification of Diseases, ninth revision, common modification (ICD-9-CM) code for gout (274.xx), a valid approach for identifying patients with gout [21]. African-Americans are usually under-represented in most qualitative research in gout with few exceptions [22, 23]; therefore a larger proportion of African-Americans were invited to participate in this study. The study participants received free parking, refreshments during the session and a payment of $30 for their participation. The Institutional Review Board (IRB, i.e. ethics review board) at the University of Alabama at Birmingham approved the study.

### Nominal group technique (NGT) sessions and analyses

#### Question assessed and NGT overview

The study team conducted patient NGT sessions/meetings lasting 1-h to understand whether and to what extent gout has an impact on relationships and sexual function. The study PI (J.S) drafted the study question with different formulations and shared separately with colleagues, researchers and patients with gout at the UAB gout clinic, who offered suggestions. After an iterative process, the study question was finalized: “How has gout affected your relationships? (think of relationship with your spouse, boy-friend or girl-friend or significant other including the effect of gout on intimacy)”. The study PI (J.S), experienced in qualitative research including NGTs [24, 25], conducted these sessions. All nominal group sessions included either women or men, except the second NGT session that included both men and women.

A nominal group technique is a variation of brainstorming where individuals come up with ideas on their own and evaluate, rank, and agree on ideas as a group; in other words it is a group process of problem identification, generation of solution/s and decision-making [26, 27]. The NGT is a variant on traditional focus group that taps the participants’ experiences, skills, views or feelings and promotes that has been used successfully in various medical settings [28–34]. One of the main differences from the focus group is that NGT allows an even participation of each participant, in contrast to possible domination by only the most active participants, and less participation by others participants. NGT also allows discussion of the problems identified.

After brief introductions by all study participants, the participants were asked if the question was clear; any/all clarification were provided before the beginning of each NGT session. A research assistant (C.G.) took notes during NGT and audio-recorded each session; an administrative assistant (D.F.) fully transcribed all the discussions verbatim, which were reviewed to ensure that the essence of discussion was captured.

#### NGT process

The NGT session consisted of the following discrete steps. They were conducted with participants seated in a large patient conference room with an oval table, and the NGT moderator and the flip chart at the head of the table. At first, each NGT participant independently quietly generated as many word or short phrases as possible in response to the question on a sheet of paper, without any discussions with other participants. This step was allocated 5–7 min depending on whether participants were still listing responses at the end of 5-min.

Each participant then nominated a single response each in a round-robin fashion, which was recorded verbatim by the NGT moderator (J.A.S.) on a flip chart in large letters visible to the group participants. Participants nominated responses until all responses were recorded. This approach prevents domination of this phase by people with a higher number of listed responses. This step took 5–10 min, depending on the number of nominated responses.

Participants then discussed and elaborated each response as a group and combined responses that seemed to be very similar, as appropriate. The NGT moderator (J.A.S.) ensured that all NGT participants participated actively in the discussion of the responses. This step took 30–40 min.

Finally, all participants identified and rank-ordered the three responses deemed important with votes from 1 (important) to 3 (most important) on index cards, 3 votes being the highest rank score. This was done by placement of colored dots on a card where participants listed their three top ranked responses (three dots for the most important concern indicating 3 votes), which were collected by the moderator or the research assistant. Scores were added from each NGT participant and the NGT moderator placed scores next to the listed responses on the flip-chart. A rank-order of nominated responses was created for each nominal group based on total scores by NGT participants, with the highest score corresponding to the top rank.

The number of nominal groups identifying responses with high relative rank ordering was analyzed. Transcriptions were examined to confirm that all main statements made relative to each response (discussions directly connected, etc.) were captured and led to the creation of a comprehensive list of statements. Responses from each NGT were compared to determine overlap, to ensure that nominal groups were performed until saturation, which was defined as the emergence of no new themes/responses.

#### Analyses

For each nominal group, an aggregate total score was calculated for listed responses on the flip chart and the ranking was determined based on the total scores from all participants, highest score being the top rank and next highest score being the 2nd ranked response/concern. The moderator calculated score for each concern. The scores were double-checked by the study coordinator (C.G.) to ensure accuracy. We examined the top ranked and top five ranked responses from each nominal group in an overall analysis across all nominal groups and presented the frequency with which each concern appeared among the top and top five responses. In addition, we also compared the total scores for the responses across all nominal groups and presented these data as a figure as a proportion of all votes, i.e. a grand total score across all nominal groups (equals 6-times the number of voting participants).

## Results

### Study participant characteristics

Fourteen nominal groups with 44 patients with gout were conducted, and saturation of themes was achieved. The mean age was 61.7 years (standard deviation [SD], 12.2; range, 40 to 83 years), 50% were men, 68% were African-American, 43% were retired and 48% were currently married (Table 1). Seven groups consisted of men only and six consisted of women only; one group had men and women. The mean duration of gout was 11.8 years (SD, 11.8) (Table 1). Seventy-nine percent of participants were using allopurinol (with/without colchicine, NSAIDs or prednisone), 15% were using febuxostat, and 5% were using only pain medications.

**Table 1:** Demographics of nominal group participants (*n* = 44)

	N (%), unless otherwise specified
Age in years, mean (SD)	61.7 (12.2)
Sex, male (%)	22 (50%)
Race/ethnicity	
White	14 (32%)
African-American	30 (68%)
Education level	
High School graduate	13 (29%)
Some college or technical/vocational training	10 (23%)
College Degree: Bachelors and beyond	21 (48%)
Marital Status	
Divorced	8 (18%)
Married	21 (48%)
Separated	3 (7%)
Single	6 (14%)
Widowed	6 (14%)
Employment status	
Employed	4 (9%)
Homemaker	4 (9%)
Out of work	5 (11%)
Retired	19 (43%)
Self-employed	3 (7%)
Unable to work	9 (20%)
Disease duration in years^a^, mean (SD)	11.8 (11.8)
Current medications to treat gout^b^	
Allopurinol (with or without prednisone)	16 (36%)
Allopurinol + colchicine (with or without pain medication)	17 (41%)
Allopurinol + colchicine + prednisone (with or without pain medication)	1 (2%)
Pain medications (NSAIDs or narcotics) with or without prednisone	2 (5%)
Allopurinol + febuxostat	1 (2%)
Febuxostat (with or without prednisone)	3 (8%)
Febuxostat + colchicine + prednisone + narcotics	2 (5%)
None	1 (2%)
Current use of natural supplements for gout^b^	
None	23 (53%)
Cherry extract or concentrate	3 (7%)
Cherry juice	10 (23%)
Multivitamin or Vitamin B or Vitamin D	7 (17%)
Number of gout flares in the last 6 months^b^	
None	17 (39%)
One	3 (8%)
Two	10 (23%)
Three to five	5 (12%)
Six or more	8 (18%)

^a^2 participants or ^b^1 participant each did not respond to these questions; Percentages are rounded off, so may not add up exactly to 100%; NSAIDs, non-steroidal anti-inflammatory drugs; SD, standard deviation

### Themes from the NGT

**Fig. 1:**
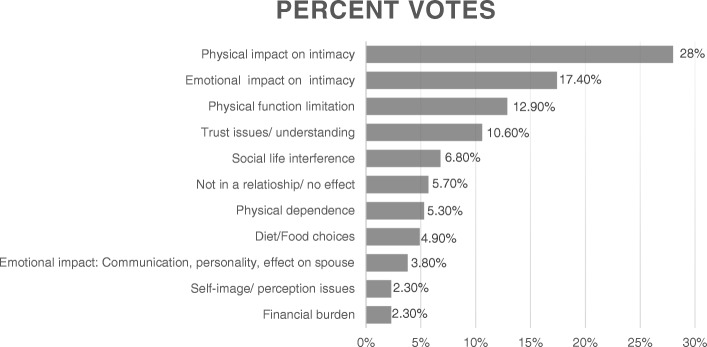
Top themes/responses of people with gout regarding its effect on the relationship with spouse or significant other. The figure shows aggregated top 11 themes/responses related to the effect of gout on relationships and intimacy. These responses accounted for 100% of the weighted votes

Various responses from participants mapped to 7 key concepts as described below. The top themes/responses from each group are listed in Table 2 with their ranking, with themes and subthemes in Table 3 with few representative quotes. Additional details of participant votes/ranking and concerns are provided in Fig. 1 (Fig. 1 shows top responses across all nominal groups by the total number score/vote for each concern, as a proportion of all votes) and Additional file 1 (Additional file 1 provides study participant nominated responses, the concept they map to along with patient quotes). The top 5 responses accounted for 75% of all votes and included physical or emotional impact on intimacy, disability, trust issues/understanding and social life interference (Fig. 1).

**Table 2:** Number of nominal groups with relative ranking of each major concern/theme

	Among male nominal groups (*n* = 7)	Among female nominal groups (*n* = 6)	All nominal groups (*n* = 14)	Among male nominal groups (*n* = 7)	Among female nominal groups (*n* = 6)	All nominal groups (*n* = 14)
	Top concern			Among top 5 concerns		
A1. Physical impact on intimacy	4	4	8	6	4	11*
A2. Emotional impact on intimacy	1	1	2	5	4	9
B1. Physical function limitation	1	1	2	3	3	6
B2. Physical Dependence	0	0	0	2	1	4*
B3. Social life interference	0	0	0	3	2	6*
C. Trust issues/understanding	1	0	2	2	1	4*
D. Self-image/perception issues	0	0	0	1	1	2
E. Diet/food choices	1	0	1	3	1	4
F. Financial burden	0	1	1	1	1	2
G. Emotional impact: communication, personality changes, effect on self/spouse	0	0	0	4	1	5
H. Not in a relationship/no or positive effect	0	0	0	2	1	3

There were 7 male only, 6 female only and 1 male and female combined nominal group

In two groups, two concerns each tied for the top rank score, therefore, there are 16 top ranked scores

*Includes one nominal group with males and females that ranked this concern in top five

Theme A consisted of Physical (A1) or emotional impact (A2) of gout on intimacy

Theme B consisted of Physical function limitation (B1), physical dependence (B2), or Social Life interference/limitation (B3)

In some nominal groups, all votes were given to < 5 concerns/themes, therefore the total rank sum for top 5 concerns adds up to less than 70

#### Physical or emotional impact on intimacy

1. Physical impact on intimacy: 11 of the 14 nominal groups ranked this among the top 5 responses, and eight nominal groups ranked it as the top concern. Patient-nominated responses, and the themes and subthemes they mapped to, are shown in Table 3**,** with illustrative quotes. Gout led to a reduction in the frequency of sexual activity. Some people “lost relationships over gout” and others were unable to be in a relationship due to gout, since their partner did not understand the pain/suffering from gout and/or did not want to be in a relationship that required them to take this kind of responsibility.
2. Emotional impact on intimacy: Nine of the 14 nominal groups ranked this among the top 5 responses; it was the top ranked concern in two nominal groups. Patient-nominated responses, and the themes they mapped to, are shown in Table 3.

**Table 3:** List of themes and subthemes from all nominal groups combined, with representative quotes/responses which are presented in bullets below each theme/subtheme

Theme/subtheme	
*A. Physical or emotional impact on intimacy*	
A1. Physical impact on intimacy: “I had	
• Difficulty with sexual activity due to gout flare pain, gout attack makes it impossible; Your whole body is to break in half if you try to be intimate”; • Lack of movement due to joint pain during gout flare; • No feeling, can’t feel sex during a flare; • Inability to maintain personal hygiene, making people feel less attractive about self; • All body pain, which made even touch to the body painful; • To lie down in the quiet room in the bed; • Inability to perform sexually when in pain; • Less desire to have sex; • Diarrhea and gas with colchicine, which was embarrassing during intimacy with spouse; • Felt sleepy with narcotic pain medication; and • Sleeping separate (in different beds or different rooms) from the spouse during a flare, for the fear of pain exacerbation with light touch.”	
A2. Emotional impact on intimacy: “I felt	
• Emotional stress due to joint pain; • Exposing I was vulnerabilities to spouse, which had a negative effect; • Spouse wasn’t aggressive towards intimacy due to my gout; • Emotional vulnerability due to “male ego” and inability to be intimate during a gout flare; • Embarrassed when my husband had to help me with personal hygiene; • Depression, anger, frustration interfering with intimacy, gout makes you mean; • That I stayed in a bad mood; • Inferior due to gout; • Depressed due to no inability to be intimate due to gout; • Emotional fragility, with my first attack, I cried like a baby; and • Gout impacted sexual desire.”	
*B. Disability/dependence Interfering with Social Life and Intimacy*	
B1. Disability: “I had	
• The inability to keep up physically with boy-friend in routine and recreational activities; • Physical disability, requiring the use of assistive devices; • Difficulty in helping wife with household chores during flare; and • The need to adjust life around the attack.”	
B2. Physical dependence: “I had	
• Total dependence on wife during a flare; • To depend on my husband/significant other; • Hospitalization for the severe pain, which was later diagnosed as a gout flare; • My spouse carry me around the house and up and down the stairs; • Gout flares that kept me in bed two to three times a month; • Inability to walk at all; and • to use crutches to walk when the flare hit.”	
B3. Limitation of Social Life activities: “I had difficulty	
• With the ability to plan events; • Going to football games or movies together with spouse; • Doing usual social activities, such as going to the bar, or a concert; • Going places due to gout pain; • Interacting with peers; • Missing church events such that spouse had to go alone; • And had to quit going to the church due to gout; • Maintaining the routine of going out for dinner due to gout pain, “a lot of times, everything stops”; and • Driving to important events due to flares, and my spouse had to drive.”	
*C. Trust issues/ understanding by spouse or significant other: “I noted*	
• Less understanding with my spouse; • Partner not taking time to understand how gout affected me; • That if the relationship was new, spouse wouldn’t understand how gout was affecting me; and • That gout made me ill-tempered with my spouse.”	
*D. Problem with Self-image and perception by partner*	
• “When I first had gout 15 years ago, I was in late 30s and I could not walk, holding on to walls; I could not drive – there was nothing going on; and • I was in a relationship and could not carry on the relationship; I couldn’t stand a sheet on my foot.”	
*E. Restricted Diet/Food choices negatively impacting the relationship*	
• “It affects a lot; • Food choices change – no shellfish, seafood; and • Places we could not eat and go out due to gout.”	
*F. Treatment-related Financial Burden stressing relationship*	
• “Gout affected income and us; • It’s stressful for the relationship; and • Medications are expensive even with insurance.”	
*G. Emotional Impact- communication, personality changes, effect on self or spouse*	
• “Women are nurturers and she could not resolve how to help me with my pain; • She wanted to find a solution for me, just couldn’t; and • She goes with me to the doctor – She is my snitch to the doctor; we had quite a different view-point about treatments, now we have a shared viewpoint: For years, I declined any medication treatment, I doctored myself- you know you go on the Internet, self-diagnose and treat yourself; it was a difficult obstacle for me to overcome.”	
*H. Not in a relationship currently/ No or positive effect on relationship*	
• "I don’t have a personal relationship; • If you are in a spiritual relationship, you can still cope; and • I knew its limitations before getting mine; It drew us closer together."	

#### Disability/dependence interfering with social life and intimacy

1. Disability: Six of the 14 nominal groups ranked this among the top 5 responses; it was the top ranked concern in two nominal groups.
2. Physical dependence: Four of the 14 nominal groups ranked this among the top 5 responses.
3. Limitation of social life activities: Six of the 14 nominal groups ranked this among the top 5 responses.
  Patient-nominated responses, and the themes and subthemes they mapped to, are shown in Table 3.

#### Trust issues/understanding by spouse or significant other

Four of the 14 nominal groups ranked this among the top 5 responses; it was the top ranked concern in two nominal groups. Patient-nominated responses, and the themes and subthemes they mapped to, are shown in Table 3**.** One of the four nominal group that ranked this concern high indicated that gout helped improve the understanding with their spouse, since it allowed them to talk about their pain to someone. The other three groups indicated that gout lead to less understanding and significant trust issues with their spouse.

#### Problem with self-image and perception by partner

Two of the 14 nominal groups ranked this among the top 5 responses. People with gout had issues with perception of self, and they felt older than their age, due to limitations related to gout, “Gout sometimes makes me feel like an old man in the relationship”.

#### Restricted diet/food choices negatively impacting the relationship

Four of the 14 nominal groups ranked this among the top 5 responses. People watched what they ate and where they went for dinner, in order to avoid foods that flared up their gout. Spouses had to play an active role in helping people avoid the foods that triggered gout attacks, and also change their own diets, not by choice. Participants indicated that restricted food choices for the couple negatively influenced their relationship with spouse/significant other.

#### Treatment-related financial burden stressing relationship

Two of the 14 nominal groups ranked this among the top 5 responses. People spent money on the care of gout, on expensive medications and hospital stay costs, which made it difficult to take vacation with spouse. Gout led to difficulty with employment, which affected income, and put a financial strain on personal relationships.

#### Emotional impact- communication, personality changes, effect on self or spouse

Five of the 14 nominal groups ranked this among the top 5 responses. People perceived personality change due to severe pain of gout and that they were short-tempered and often snapped at their spouse. These often led to miscommunication and misunderstanding. Several people also reported that gout affected their spouse’s behavior in a negative way, making them worried, aggravated and sometimes blaming themselves. Gout led to a change in social role in several relationships, where husband had to learn to share household chores with the wife suffering from gout.

#### Not in a relationship currently/ no or positive effect on relationship

Three of the 14 nominal groups ranked this among the top 5 responses, of whom two nominal groups had people who had not been in a relationship since the diagnosis of gout, and therefore were unable to assess whether it would or would not have an effect on relationships. Gout was not the reason to not be in a relationship. In one nominal group, people noted that they had had a good control of gout (with medications and diet) with infrequent flare/s, and since their gout was infrequent, they had not had any impact of gout on their relationship at all.

**Fig. 2:**
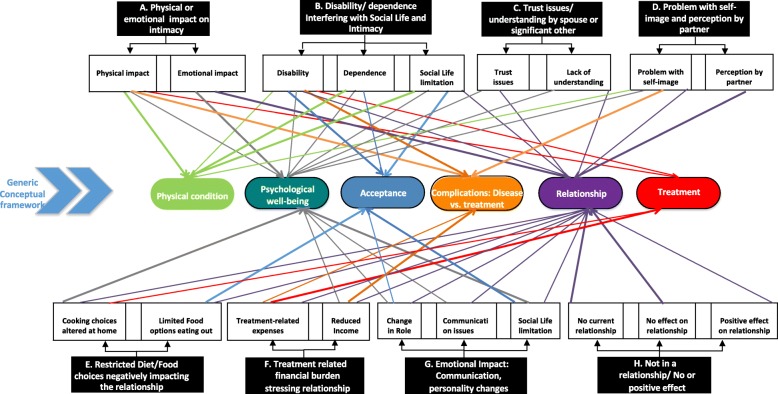
Responses from NGT participants regarding the impact of gout on their relationships with spouse or significant other. The figure shows the associations of key themes (black shaded boxes) and contributing categories (clear boxes) to various constructs from a generic conceptual framework (colored ovals)

Figure 2 shows the main themes/subthemes derived from patient-nominated responses, and their mapping to the generic conceptual framework proposed by Verschuren et al. [14].

### Effect of gender on intimacy/sexual function responses

The number of nominal groups ranking the following overall top responses were similar between male and female nominal groups: (1) physical impact on intimacy, 4/7 male vs. 4/6 female; (2) emotional impact on intimacy, 1/7 male vs. 1/6 female; and (3) disability, 1/7 male vs. 1/6 female; and (4) trust issues/understanding, 1/7 male vs. 0/6 female.

Potentially more male than female nominal groups ranked the following among the top five responses, (1) emotional impact on communication and personality change, 4/7 male vs. 1/5 female and (2) restricted diet/food choices negatively impacting the relationship, 3/7 male and 1/6 female nominal groups.

## Discussion

This formative research showed that gout had a significant impact on relationships and sexual activity of people with gout. The physical impact of gout on intimacy was the top-ranked concern across all nominal groups by the number of votes (28%), as well as the number of nominal groups ranking it the highest, eight of the 14 nominal groups. Gout impacted relationship and intimacy in both men and women with gout. With minor exceptions, we did not note any significant differences in the top concern related to gout by patient gender. These data provide the patient perspective of the impact of gout on intimacy. Inclusion of African-Americans and women in our study sample makes these findings more generalizable.

Studies in other inflammatory arthritides reported that pain, physical disability, joint deformity and concomitant depression were associated with sexual dysfunction [17–20]. Studies in men with gout showed that gout was associated with more erectile dysfunction [8–10]. No data on sexual impact are available for women with gout. Our formative study advances the field by providing quantitative and qualitative data on sexuality in people with gout, and including women in our study, both first to our knowledge.

Data from our nominal groups mapped to the generic conceptual framework proposed by Verschuren et al. [14]. This framework considers sexuality to be a multifaceted phenomenon, affected by organic, hormonal, and psychosocial factors, and that chronic illness involves physical symptoms and psychosocial stressors. Several themes and subthemes mapped to the key constructs of physical condition, psychological well-being (two top-ranked themes in our nominal groups were similar – physical/emotional impact on intimacy), and relationship, in the conceptual framework [14] (Fig. 1). The current study found that disease and/or treatments impact sexual function in patients with gout. Gout is associated with a higher risk of metabolic syndrome [35], which might also contribute to sexual desire and performance in gout. Mapping our data to this framework not only provides insight into possible mechanisms of sexual dysfunction in gout, but may also form the basis of the development of interventions to address sexual dysfunction in gout.

A clinical implication of this study is that a patient-physician dialogue is necessary to assess whether or not gout is currently impacting their sexual life, and if so, better understand the effect. Considering the important contribution of sexuality to QOL [11], optimal gout management with treat-to-target strategy [36, 37] to reduce gout symptoms and flare rates can potentially reduce the impact of gout in sexual health and improve patient’s QOL. For people with refractory sexual dysfunction despite optimal gout management, referral to an expert in psychiatry or sexual health may benefit a sizeable proportion of gout patients and improve their QOL.

Considering that we invited a convenience sample of consecutive patients with gout (not people with diagnosed sexual health problems), the proportion of people reporting and discussing the impact of gout on relationships with spouse/significant other and sexual dysfunction was much higher than expected; with the exception of a few, almost everyone reported some impact. While sexual problems were a hallmark of gout flare associated severe pain, a majority of the nominal groups reported frequent sexual dysfunction due to chronic joint pain due to gout, most notably difficulty performing sexually due to gout-associated pain. The study findings demonstrate the physical impact of acute and chronic pain of gout and associated disability on intimacy and sexual function. Future studies that aim for a reduction of gout flares and chronic joint pain in gout should examine whether acute and chronic pain reduction can potentially have a positive impact on sexual activity and relationship with spouse as important patient-centered domain/outcome. Inclusion of sexual function as a secondary or exploratory outcome in clinical trials of gout will improve our understanding of the relationship of active gout symptoms and sexual dysfunction. They will also help us understand whether therapies with varying effects on inflammation and hyperuricemia differ in their ability to reduce gout’s impact on sexual function. Could a more optimal gout control (fewer gout flares, reduced joint pain) improve relationship and intimacy? Might management and optimization of associated depression have a positive impact? These hypotheses need to be tested in future studies.

Another important, novel study finding was the emotional impact of gout on intimacy. This was the 2nd top-ranked concern across all nominal groups based on the number of votes (17.4%) and two of the 14 nominal groups had this as their top concern. We are unaware of any other published studies of the emotional impact of gout on sexuality, intimacy and relationships, except our previous study where the focus was quality of life [7]. Associated depression, emotional stress, anger and frustration impact intimacy. Gout-related pain exposed patient’s vulnerabilities to their spouse that negatively affected their relationship. Patients identified several other causes of emotional impact of gout on intimacy, including the need to get help from spouse in maintaining personal hygiene. A feeling of inferiority due to the inability to be intimate also affected people with gout. Feeling of emotional fragility by both women and men with gout and of vulnerability due to “male ego” by men with gout were also reported. Patients also reported a reduced sexual desire due to gout, which might be related to associated depression and/or to concomitant metabolic syndrome associated conditions. Trust issues, disability and social life interference due to gout were of concern to the patients and ranked among the top five responses across all groups, as shown in the figure.

Some people lost relationship due to active, symptomatic gout (usually under-treated and sometimes undiagnosed/misdiagnosed) and some had difficulty getting into a relationship due to gout, demonstrating a significant effect of gout on people’s lives. To our knowledge, this has not been previously described. This finding indicates the severe, disruptive effect of inadequately controlled gout. Interestingly, one nominal group with gout under good control with few/no flares indicated that gout had not affected their relationship. Additionally, a few people in two nominal groups had not had a relationship since the diagnosis of gout, and therefore could not assess its effect on relationships; the choice of not being in a relationship was not related to gout (different from people described at the beginning of the paragraph). We also found that in rare instances, gout had no effect or a positive impact participant’s relationship. Participants attributed this positive experience to an understanding spouse, a strong relationship with spouse prior to the disease appearance, and infrequent gout flares.

Study findings must be interpreted with caution considering study limitations. Findings may not be generalizable to all Americans with gout, since this was a single center study of people previously evaluated for gout at a community-based clinic, and the nominal groups were conducted in English only. Due to the sensitive nature of the question, it is possible that people did not share the most intimate aspects of their relationships; some responses may have been missed. Assessment of possible solutions to the prioritized problems by the patients would have required another 1–1.5 h of nominal group discussions and were not assessed due to limited time. This is an important research agenda that needs to be addressed with future studies. Interpretation of findings by a single researcher is another study limitation. However, phrasing and nomination of responses, addition of details to the participant-nominated responses, the decision to group or ungroup responses, voting and ranking are all done by the nominal group participants, not the moderator. Therefore, it is unlikely that the number of researchers involved had any impact on the quantitative aspect of the NGT, which were the main study findings.

Study strengths were the inclusion of women and African-Americans, the achievement of data saturation, and the study cohort demographics being similar to other studies of gout populations.

## Conclusions

In conclusion, this formative study assessed the effect of gout on relationships with spouse/significant other and sexual function. In a community-based outpatient clinic sample of people with gout, we found that gout affected relationships with spouse and sexual function quite commonly, and in several ways. A major impact was related to the acute and chronic joint pain and the associated disability, with physical and emotional impact on intimacy being the highest ranked responses. Study participants also noted loss of trust with spouse, loss of relationship, and significant social life impact due of gout. This study advances our knowledge of the true impact of gout on people’s sex lives. This study also brings to light an aspect of patient suffering, previously not recognized or well-described. The healthcare providers should discuss the impact of gout on relationships with patients with gout during clinic visits and address it appropriately. A reversal of a negative impact of gout on intimacy with appropriate disease control can serve as a positive reinforcement for some patients and might help to increase treatment and medication adherence.

## Additional file

Additional file 1: Study participant nominated responses (numbered), the concept they map to (in parenthesis) along with patient quotes (individually bulleted items) and the score/votes each received from the nominal group participants in the final voting phase. (DOCX 79 kb)

## Abbreviations

ER: Emergency room
NGT: Nominal Group Technique
UAB: University of Alabama at Birmingham
ULT: Urate-lowering therapy

## References

[1] Smith E, Hoy D, Cross M, Merriman TR, Vos T, Buchbinder R, Woolf A, March L (2014). The global burden of gout: estimates from the global burden of disease 2010 study. Ann Rheum Dis.

[2] Elfishawi MM, Zleik N, Kvrgic Z, Michet CJ, Crowson CS, Matteson EL, Bongartz T (2018). The rising incidence of gout and the increasing burden of comorbidities: a population-based study over 20 years. J Rheumatol.

[3] Chandratre P, Roddy E, Clarson L, Richardson J, Hider SL, Mallen CD (2013). Health-related quality of life in gout: a systematic review. Rheumatology (Oxford).

[4] Singh JA, Strand V (2008). Gout is associated with more comorbidities, poorer health-related quality of life and higher healthcare utilisation in US veterans. Ann Rheum Dis.

[5] Roddy E, Zhang W, Doherty M (2007). Is gout associated with reduced quality of life? A case-control study. Rheumatology (Oxford).

[6] Zhu Y, Pandya BJ, Choi HK (2012). Comorbidities of gout and hyperuricemia in the US general population: NHANES 2007-2008. Am J Med.

[7] Singh JA (2014). The impact of gout on patient's lives: a study of African-American and Caucasian men and women with gout. Arthritis Res Ther.

[8] Schlesinger N, Radvanski DC, Cheng JQ, Kostis JB (2015). Erectile dysfunction is common among patients with gout. J Rheumatol.

[9] Hsu CY, Lin CL, Kao CH (2015). Gout is associated with organic and psychogenic erectile dysfunction. Eur J Intern Med.

[10] Abdul Sultan A, Mallen C, Hayward R, Muller S, Whittle R, Hotston M, Roddy E (2017). Gout and subsequent erectile dysfunction: a population-based cohort study from England. Arthritis Res Ther.

[11] McInnes RA (2003). Chronic illness and sexuality. Med J Aust.

[12] Basson R, Rees P, Wang R, Montejo AL, Incrocci L (2010). Sexual function in chronic illness. J Sex Med.

[13] Nusbaum MR, Hamilton C, Lenahan P (2003). Chronic illness and sexual functioning. Am Fam Physician.

[14] Verschuren JE, Enzlin P, Dijkstra PU, Geertzen JH, Dekker R (2010). Chronic disease and sexuality: a generic conceptual framework. J Sex Res.

[15] Jensen SB (1992). Sexuality and chronic illness: biopsychosocial approach. Semin Neurol.

[16] Hill J, Bird H, Thorpe R (2003). Effects of rheumatoid arthritis on sexual activity and relationships. Rheumatology (Oxford).

[17] Esteve E, Maccari F, Delavierre D, Vicariot C, Charles B, Marty M, Lespessailles E (2018). Preliminary development of a questionnaire assessing the impact of psoriasis and psoriatic arthritis on patient's perception of sexuality. Medicine (Baltimore).

[18] Kobelt G, Texier-Richard B, Mimoun S, Woronoff AS, Bertholon DR, Perdriger A, Maugars Y, Combe B (2012). Rheumatoid arthritis and sexuality: a patient survey in France. BMC Musculoskelet Disord.

[19] Kraaimaat FW, Bakker AH, Janssen E, Bijlsma JW (1996). Intrusiveness of rheumatoid arthritis on sexuality in male and female patients living with a spouse. Arthritis Care Res.

[20] Herstein A, Hill RH, Walters K (1977). Adult sexuality and juvenile rheumatoid arthritis. J Rheumatol.

[21] Singh JA, Hodges JS, Toscano JP, Asch SM (2007). Quality of care for gout in the US needs improvement. Arthritis Rheum.

[22] Te Karu L, Bryant L, Elley CR (2013). Maori experiences and perceptions of gout and its treatment: a kaupapa Maori qualitative study. J Prim Health Care.

[23] Lindsay K, Gow P, Vanderpyl J, Logo P, Dalbeth N (2011). The experience and impact of living with gout: a study of men with chronic gout using a qualitative grounded theory approach. J Clin Rheumatol.

[24] Singh JA: Challenges with gout treatment faced by patients: a qualitative study (in press). J Clin Rheumatol 2014. 10.1097/RHU.0000000000000091 PMC4169366 24662562

[25] Singh JA. Research priorities in gout: the patient perspective (in press). J Rheumatol. 2014. 10.3899/jrheum.131258 PMC4169368 24585526

[26] Nominal Group Technique: Definition and Example. https://study.com/academy/lesson/nominal-group-technique-definition-example.html. Accessed 30 Jan 2019.

[27] Nominal Group Technique. https://en.wikipedia.org/wiki/Nominal_group_technique. Accessed 30 Jan 2019.

[28] Jefferson WK, Zunker C, Feucht JC, Fitzpatrick SL, Greene LF, Shewchuk RM, Baskin ML, Walton NW, Phillips B, Ard JD (2010). Use of the nominal group technique (NGT) to understand the perceptions of the healthiness of foods associated with African Americans. Eval Program Plann.

[29] Kleiner-Fisman G, Gryfe P, Naglie G (2013). A patient-based needs assessment for living well with Parkinson disease: implementation via nominal group technique. Parkinsons Dis.

[30] MacLachlan M (1996). Identifying problems in community health promotion: an illustration of the nominal group technique in AIDS education. J R Soc Health.

[31] Miller D, Shewchuk R, Elliot TR, Richards S (2000). Nominal group technique: a process for identifying diabetes self-care issues among patients and caregivers. Diabetes Educ.

[32] Pastrana T, Radbruch L, Nauck F, Hover G, Fegg M, Pestinger M, Ross J, Krumm N, Ostgathe C (2010). Outcome indicators in palliative care--how to assess quality and success. Focus group and nominal group technique in Germany. Support Care Cancer.

[33] Pena A, Estrada CA, Soniat D, Taylor B, Burton M (2012). Nominal group technique: a brainstorming tool for identifying areas to improve pain management in hospitalized patients. J Hosp Med.

[34] Redman S, Carrick S, Cockburn J, Hirst S (1997). Consulting about priorities for the NHMRC National Breast Cancer Centre: how good is the nominal group technique. Aust N Z J Public Health.

[35] Choi HK, Ford ES, Li C, Curhan G (2007). Prevalence of the metabolic syndrome in patients with gout: the third National Health and nutrition examination survey. Arthritis Rheum.

[36] Khanna D, Fitzgerald JD, Khanna PP, Bae S, Singh MK, Neogi T, Pillinger MH, Merill J, Lee S, Prakash S (2012). 2012 American College of Rheumatology guidelines for management of gout. Part 1: systematic nonpharmacologic and pharmacologic therapeutic approaches to hyperuricemia. Arthritis Care Res (Hoboken).

[37] Kiltz U, Smolen J, Bardin T, Cohen Solal A, Dalbeth N, Doherty M, Engel B, Flader C, Kay J, Matsuoka M (2016). Treat-to-target (T2T) recommendations for gout. Ann Rheum Dis.

