# Supplementary material for: Gout and sexual function: patient perspective of how gout affects personal relationships and intimacy
Source: BMC Rheumatol. 2019 Feb 28;3:8. doi: 10.1186/s41927-019-0056-9 (PMC6396531; doi:10.1186/s41927-019-0056-9)
Supplement: Supplementary file 1 — Study participant nominated responses (numbered), the concept they map to (in parenthesis) along with patient quotes (individually bulleted items) and the score/votes each received from the nominal group participants in the final voting phase. (DOCX 79 kb) [file 41927_2019_56_MOESM1_ESM.docx]

**Additional File 2.** Study participant nominated responses (numbered), the concept they map to (in parenthesis) along with patient quotes (individually bulleted items)

| **NGT #1 9/18/17 8:00 AM 2Male 1African-American 1White** | |
| --- | --- |
| 1. **Unable to have a romantic evening due to pain** (A. Physical Impact-Intimacy)  - If your upper and lower extremities are hurting it’s seldom that you can have a good evening with your wife - Your whole body is to break in half if you try to be intimate | **Score: 6** |
| 1. **Sometimes gout meds make you go to the restroom, can’t perform as you’re supposed to- the sexual interaction** (A. Physical Impact-Intimacy)    - Colchicine makes you have bad gas, so embarrassing with your wife | **Score: 2** |
| 1. **Depression**/**Anger/frustration** (A. Emotional Impact-Intimacy)  - Interferes with sexual intercourse - Feeling inferior - You can be in a great mood but a depressed mood can turn you off - She’d be better off with someone normal - Keeps me in a bad mood - Can’t do what you used to do - Makes you mean - It’s the worst pain - My knee surgery wasn’t as bad - I wouldn’t wish it upon an enemy - Trouble getting pain meds and feeling like a criminal | **Score: 2** |
| 1. **Not being able to interact with peers** (B. Social life Interference)  - Things you can’t do hurts your wife emotionally; anger and frustration - Used to go watch football games together, can’t do that - Miss so many church events, I quit church band due to gout - Being able to plan events | **Score: 1** |
| 1. **Financial burden** (F. Treatment-related Financial Burden stressing relationship)  - Trickle down affect when you spend money, you can’t take your wife out for a vacation together   - Was hiking and caving with her before gout hit me; no more - Stress that it puts on her affects her in negative ways | **Score: 1** |
| 1. **Gout, due to the attention it needs, can stress your wife out** (B. Physical Dependence)  - Hands swell up, wife is helping you clean your feces - I ring a little bell when I can’t move (during a flare), I think that annoys my wife - Not able to drive, can’t fasten seatbelt without help - Gout flares up and you need help to walk - Your hands are hurting and you are unable to do your AM care - Having wife to help you get up; I have a small wife, she has to struggle to do that- I am more needy now in our relationship; I always want to take care of her | **Score: 0** |
|  | **Total: 12** |
|  |  |
| **NGT #2 9/18/17 11:00 AM 3Female 2Male 5African-American 0White** | |
| 1. **Less understanding in couples** (C. Trust issues/Understanding)    - If you are trying to get them to understand – does that affect relationship?    - Gout made me ill-tempered      - If I am in pain, I don’t want to be bothered      - I get “fussy”      - Dependent on a person      - “Less tolerant”      - On first episode with gout, couldn’t sleep with my wife – couldn’t get along, could not have a conversation    - Everything slowed down    - My husband is very understanding – he has mild gout | **Score: 12** |
| 1. **Sexual Activity** (A. Physical Impact-Intimacy)    - Can’t get a coalition    - I might use it as an excuse    - At that point (with so much pain) who wants to have sex; that’s the last thing on my mind | **Score: 8** |
| 1. **Made me dependent** (B. Physical Dependence)    - Was so painful it put me in the hospital, they were talking about knee replacement, then they found out I had gout    - I had flare, couldn’t move, she literally had to pick/ carry/me up and down the stairs    - Had to help me put on socks    - Hard to dress up and do basic stuff    - Dependence is bad for basic stuff – feel like a burden    - Emotional aspect of relationship | **Score: 6** |
| 1. **A lot of times, everything stops** (B. Social life Interference)    - You just want that pain to stop    - You don’t want to go out      - Going out stops, she may want to go out    - Household Chores are a problem, the responsibility shifts to your spouse | **Score: 3** |
| 1. **Participate in events – can’t do that anymore** (B. Social life Interference)    - Can’t go to movies    - My friend with gout – “broken up now”, he is not in relationship anymore    - (My brothers’) Girlfriend didn’t want to deal with gout, diabetes and related issues, brother had 2 relationships that ended because of gout      - May not understand how much pain he was in?      - After every attack, they (Girlfriends) got less interested. He really loved this lady, but the relationships ended. | **Score: 1** |
|  | **Total: 30** |
|  |  |
| **NGT #3 10/16/17 8:00 AM 2Female 2African-American** | |
| 1. **Sometimes you want to but you’re not able to be intimate – now you are depressed because you can’t** (A. Emotional Impact-Intimacy)    - When you have an illness – depression is already there    - I become depressed when I know my illness is affecting my relationship    - I get depressed because I couldn’t be there for him    - When I have a flare I like sitting up in a chair rather than be in the bed; My friend wants me to lay down next to him if I don’t, it takes away from the relationship    - When in pain, don’t want to be bothered | **Score: 4** |
| 1. **When I am having a flare, the bottom line is that I don’t want to be intimate** (A. Physical Impact-Intimacy)    - I don’t want to be intimate, period.      1. Pain      2. Lack of movement    - I don’t want anyone even in bed, near me or touching me      1. During the flare – you just want to sleep    - The bottom-line is I don’t want to move when it flares- it’s in my knees, toes      1. I have to prop-up my feet: It’s just pain, just want to scream out    - When I am in pain, I can’t feel sex because that’s the last thing on my mind      1. Trying to get some rest | **Score: 3** |
| 1. **I am not able to perform my best personal hygiene during a flare, the way I usually can** (A. Physical Impact-Intimacy)    - I can’t shower well    - Hygiene wasn’t up to normal – I didn’t like that- You don’t feel attractive | **Score: 2** |
| 1. **When it flares up I depend on my friend more** (B. Physical Dependence)    - All the time is spent in getting care for my gout which affects his work and affects his relationship with children      1. It makes me feel bad, I feel that I am putting pressure on my relationship with him      2. Once I fell off wheelchair, I couldn’t call anyone my son got me off floor    - Affects my relationship with my grandchildren (16, 14, 19) – I am their caregiver      1. He has stepped up to help me; He has to drop everything he is doing – how much can he take? | **Score: 2** |
| 1. **When I take a pain medication (Lortab) I am woozy – You are not aware enough – not interested your mind is not focused on that (intimacy)** (A. Physical Impact-Intimacy)    - Medication just puts you to sleep – “I am out” | **Score: 1** |
| 1. **My partner didn’t understand gout and why I didn’t want to be bothered** (C. Trust issues/Understanding)    - He didn’t take the time to understand – when I had surgery it was visible, he understood; but gout, he couldn’t understand | **Score: 0** |
|  | **Total: 12** |
|  |  |
| **NGT #4 10/16/17 10:00 AM 4Male 4White** | |
| 1. **Accommodation/Understanding is primarily by the partner** (C. Trust issues/Understanding)    - If you were just married she wouldn’t understand, it would be a problem    - Suffered from gout on and off X 50 years, don’t know if it has affected relationships off and on    - An understanding partner is a big plus    - The length of relationship matters- we know each other and care    - Always had a cane, or a walker, so I didn’t lose any work (due to flares)    - Wife was very patient    - My wife is an RN – she is very understanding – Flare up was short and infrequent - wasn’t long enough for abstain from sexual activity    - Don’t think sexual activity is the focal point of the relationship; My wife is very understanding    - Don’t believe it has affected understanding between us – I have had gout for 51 years    - Both wives were very understanding | **Score: 11** |
| 1. **Pain during the flare eliminates any desire for anything other (intimacy) than trying to find relief** (A. Physical Impact-Intimacy)    - Don’t think anybody can perform when in so much pain    - Such a random onset, I couldn’t tell when it was coming    - Comes back in my toes, heel, foot: I was hospitalized; “It hurts like hell”, so it bothers you – hospitalized once for gout    - When I was being diagnosed, doctor took samples from fingers – Don’t know how much hand problem is gout | **Score: 6** |
| 1. **Stress/aggravation of frequent flares** (A. Emotional Impact-Intimacy)    - Very frustrated; can’t be next to wife    - “Just cut my leg off”    - Can’t even bend your leg to put on the socks    - Had a trip, couldn’t go for it | **Score: 6** |
| 1. **Was diagnosed with gout and Pseudogout 25 years ago; had routine attacks – were debilitating and affected every part of the life** (B. Disability)    - Duration affects me and my significant other- you have to adjust your life around the attack    - Attacks were frequent and severe, but my wife is an RN    - I was unmarried, in some ways it did; I didn’t want to go bar-hopping limping – A man is not attractive limping in and out of a bar | **Score: 1** |
|  | **Total: 24** |
|  |  |
| **NGT #5 10/18/17 10:30 AM 3Female 2African-American 1White** | |
| 1. **During the flare, didn’t want to touch (pain)** (A. Physical Impact-Intimacy)    - Flare is all over the body, “please don’t touch me”    - When you are hurting, you can’t stand your partner – pain and the emotional part    - Really bad pain, it will make you angry    - During the flare, I don’t want to ask for help, or assistance but I need it; I am independent, living by self    - When arm is flared, need to ask help with personal hygiene to wipe myself- it’s a pride thing, it impacts self-respect.    - Spending less time with spouse | **Score: 7** |
| 1. **Unable to do everyday activities** (B. Disability)    - During a gout flare with friends and family such as park    - “We will just do this because she can’t come with us”    - I need to have air “off” during flare; they (my family) all just go outside    - Be like to be by myself when it flares; Spouse may think you are ignoring them    - He pushed me to make me walk | **Score: 6** |
| 1. **During the flare, I had to sleep in a separate room which interfered with intimacy** (A. Physical Impact-Intimacy)    - Had to sleep in bed closer to rest room and protect my knee from getting hit by significant other | **Score: 3** |
| 1. **I like to just lie down in my bed all the time during the flare** (A. Physical Impact-Intimacy)    - So you won’t hurt    - You feel stress/anger, you just say “no” – interferes with sex | **Score: 1** |
| 1. **When dating someone new it’s hard to explain why you walk slow or can’t do something** (B. Disability)    - I am 5 years younger than this person I was dating, I was having pain, just couldn’t keep up – unable to keep up physically for routine activities and recreational activities | **Score: 1** |
| 1. **My husband would get angry when I would have gout attack in my knee or foot, and he didn’t understand situation** (C. Trust issues/Understanding)    - What’s wrong with you, why can’t you do this?    - Had a significant other who didn’t understand until he had gout in his toe    - Sometimes get into argument due to my pain | **Score: 0** |
|  | **Total: 18** |
|  |  |
| **NGT #6 10/19/17 12:45 PM 2Male 1African-American 1White** | |
| 1. **Gout causes me to become less mobile during a flare-up** (A. Physical Impact-Intimacy)    - I turn inward due to pain – I get quiet, I don’t want to talk    - I have few major flare-ups – Nothing is going to happen during those times    - Going out to dinner during a flare is not an option | **Score: 4** |
| 1. **Gout causes me to watch my diet and where we go to eat shellfish** (E. Restricted Diet/Food choices negatively impacting the relationship)    - She helped me avoid some foods – she keeps me on track    - I can have fights over these things – “We have had this conversation”    - We’re at dinner during vacation - you know you are not supposed to do that    - I take my medication – double up when I indulge | **Score: 3** |
| 1. **It helps me to talk about my pain to someone – brought us closer** (C. Trust issues/Understanding)    - My tolerance for pain is low – I like to talk complain about it - she kept me from getting depressed    - Knowing someone cared for my pain - she is there    - Gives us something to laugh about – to be able to vent really helps me    - She helped me get around the house    - She has changed restaurants based on what can make my gout worse | **Score: 2** |
| 1. **Gout causes me to become short-tempered/stress when I am in pain** (G. Emotional Impact-Communication)    - I don’t want to talk, then I snap | **Score: 2** |
| 1. **Gout sometimes makes me feel like an old man in the relationship** (D. Problem with Self-image/ perception)    - When I first had gout 15 years ago, I was in late 30s and I could not walk, holding on to walls; I could not drive – there was nothing going on    - I was in a relationship and could not carry on the relationship; I couldn’t stand a sheet on my foot | **Score: 1** |
|  | **Total: 12** |
|  |  |
| **NGT #7 11/1/17 8:00 AM 2Male 2 African-American** | |
| 1. **Lost a relationship over gout** (A. Physical Impact-Intimacy)    - Wife begins helpful and concerning, but some just ain’t going to stay    - Know someone who had just started relationship, she left saying “I hadn’t signed up for this”    - I had a friend who couldn’t work for 3 months, not even with a walker, I think that affected their relationship; He has to have people (home health) help him despite wife. | **Score: 3** |
| 1. **Mind is registered on the pain** (A. Emotional Impact-Intimacy)    - Can’t think about anything but your pain    - It was stressful    - Spouse had to do more things than normal – overwhelmed/stressed; Affects her work and other relationships with family, and most of all, you    - Everyone needs their own time – if you have to care for someone all the time – could get overwhelming quickly    - With my first attack, I cried like a baby | **Score: 3** |
| 1. **My friend had told me that it had kept him in bed two to three times a month** (B. Disability)    - Some had flares 2-3 times a month    - During flares, any activity becomes difficult (going out and getting up)    - I couldn’t walk after my surgery due to gout, not because of the surgery, but because of my gout (flaring); That was a whole week    - Didn’t think anything could hurt as bad as gout    - Flare up can be any moment    - I was on vacation, flare hit, then I was on crutches | **Score: 2** |
| 1. **Some have told me that it affects their sex life and sex drive** (A. Physical Impact-Intimacy)    - For sex life, it goes back to your mate    - For 6 months, couldn’t walk – used a walker    - Flare up occurred on our anniversary, sex wasn’t even a thought; It’s not important at the time    - When flare-ups arrive, sex decreased    - It affects intimacy    - It all depends in your mate    - It would be hurting – anything you do | **Score: 2** |
| 1. **It made my wife worry more** (G. Emotional Impact-Communication)    - She just wanted me to be fixed    - We have 4 kids; when I can’t move I become the 5^th^ kid | **Score: 1** |
| 1. **Wife was most supportive during flares trying to help me be independent – physical dependence** (B. Physical Dependence)    - I do a lot of stuff myself – with flares I couldn’t drive- she’d bring me around    - It can affect relationships – if you have a spouse depend on you to do everything – affects negatively    - If you are the breadwinner, spouse relies on you | **Score: 1** |
|  | **Total: 12** |
|  |  |
| **NGT #8 11/1/17 10:30 AM 3Female 3 African-American** | |
| 1. **The Physical Impact** (A. Physical Impact-Intimacy)    - My sister in law has gout flare, medicine precludes them to have relationship    - She is the caretaker; when she has gout both of them are sitting looking at each other and that’s all they can get    - When she has gout flare, she becomes “mean”    - If my knee is hurting, I don’t want to see you    - My grandfather has gout they never discussed their relationship, but he was in a lot of pain during flares; He didn’t believe in doctors, took Tylenol and cherries – it would take a week    - My brother-in-law had gout, who is passed away – when he had flares he couldn’t do much of anything      1. Other things weren’t happening      2. He was also diabetic and had cancer and was dialysis      3. He was a very active person socially, he’d just sit and look at TV    - He as sleeping on a sofa, not going to bedroom | **Score: 6** |
| 1. **I have a male cousin with gout – affected him mentally and he doesn’t cope that well** (A. Emotional Impact-Intimacy)    - Once there is a flare, He complained a lot, used to say “I will be stuck with it now”. He is not able to cope with it. He is single, and he is in and out of relationships. Before he got gout, he used to be fun to be with; not any more | **Score: 5** |
| 1. **I am not in a personal relationship at this time** (H. Not in relationship/No or positive effect on relationship)    - I don’t have a personal relationship    - If you are in a spiritual relationship, you can still cope    - I knew its limitations before getting mine; It drew us closer together    - At times, may push you closer, It depends on your spouse | **Score: 4** |
| 1. **If you are not in a spiritual relationship it can end your relationship** (A. Physical Impact-Intimacy)    - Gout or flares | **Score: 3** |
|  | **Total: 18** |
|  |  |
| **NGT #9 11/1/17 12:45 PM 4Male 2White 2African-American** | |
| 1. **It really hasn’t affected my relationship at all** (H. Not in relationship/No or positive effect on relationship)    - It hasn’t affected me; I had only one flare    - Mine hasn’t been bad, I have not had it bad at all    - Found out what foods to avoid, take medications regular    - Other people I talk to talk about pain, not about relationships | **Score: 8** |
| 1. **When it flares, pain slows me down** (A. Physical Impact-Intimacy)    - I can’t walk on my feet, I have to wait for it to go away    - My sister has to come to our house to help me    - When I have flare, my wife understands it    - When you have flare, you don’t want anything close to it (flared joint) | **Score: 8** |
| 1. **Interferes with social life** (B. Social life Interference)    - Can’t go to parties, socialize | **Score: 5** |
| 1. **When I have pain, I have to stay in bed for 2-3 weeks, until it goes down** (A. Physical Impact-Intimacy)    - When pain starts, I can’t do anything    - When you are in pain, you say, not tonight baby    - Pain hurts    - In pain, you can’t sleep    - Its excruciating pain; It’s awful, you don’t get any warning; nothing happens when you have gout pain | **Score: 3** |
|  | **Total: 24** |
|  |  |
| **NGT #10 11/13/2017 10:30 AM 5Female 5African-American** | |
| 1. **It was depressing at first, not knowing exactly what was wrong and the type of arthritis** (A. Emotional Impact-Intimacy)  - Affects your desire to be intimate - Puts me in a bad mood sometimes, I don’t want to be bothered or touched - Sometimes it affects your mind - Trying to make everybody happy - I am the only person in the family with it | **Score: 9** |
| 1. **Toe was getting real red – It affected me from doing a lot of things** (B. Disability)  - Kept from walking – If you have plans for the evening, you have to cancel your plans - Even if you go, you will still be hurting - I don’t go out as much, always in pain - Not knowing when is going to flare up and which “arthritis” it is - I have different illnesses, lupus, fibromyalgia and gout, not knowing its affects – one may be worse than the other - I don’t do things I used to do - I don’t want to go many places anymore | **Score: 5** |
| 1. **Ability to wear heels** (D. Problem with Self-image/ perception)  - In case you want to look good - I want to look attractive in heels; I have a lot of heels and I can’t wear them | **Score: 5** |
| 1. **He thought it only affected men and old people; he always thought I was lying** (C. Trust issues/Understanding)  - It got me angry - Spouse didn’t believe “you were just hurting all the time” - Trust issues - He may not understand your pain | **Score: 3** |
| 1. **I am separated from my husband of 7 years. When I get it in my knees, I can hardly walk** (B. Disability)  - I got gout after separating | **Score: 3** |
| 1. **I used to ache all the time. It would affect my relationship** (A. Emotional Impact-Intimacy)  - Didn’t have time to be with him (due to my gout) - I never know when I am going to have a flare-up - You are planning to do something or go somewhere; I’d be hurting - Just don’t be intimate | **Score: 2** |
| 1. **My spouse is always thinking I am hurting** (A. Emotional Impact-Intimacy)  - If he thinks you are hurting, he won’t want to be intimate - It has made him more sensitive and aware to how I feel about my pain and if I’m in pain - Overprotective: Always watched over me – sometimes I get upset - I am not in such severe pain - Negative effect on intimacy - My husband has always taken care of me when I go somewhere | **Score: 2** |
| 1. **When I use my cane, didn’t want to be seen with it** (B. Disability)  - Related back to my age - My spouse didn’t want to go out - embarrassed - Gout starts with tingling in toes … I had to use a walker and a cane | **Score: 1** |
| 1. **My father had gout and I saw that affected him, he was in a lot of pain** (B. Disability)  - My mother had to do everything – tried “homemade remedies” – affected their relationship - Sister also has gout – call each other every morning – affected them in sexual ways | **Score: 0** |
|  | **Total: 30** |
|  |  |
| **NGT #11 12/11/2017 1:30 PM 3Male 2White 1African-American** | |
| 1. **Diet is global** (E. Restricted Diet/Food choices negatively impacting the relationship)    - It affects a lot    - Food choices change – no shellfish, seafood    - Places we could not eat and go out due to gout | **Score: 6** |
| 1. **Fear of pain: Emotional** (A. Emotional Impact-Intimacy)    - The pain affected me even when it (flare) wasn’t happening    - Changes personalities    - This is the last time to go through this – you are not at a good place to be a good husband    - Emotional distress    - Cutting back on things to do with spouse due to fear of flares | **Score: 6** |
| 1. **I was blaming myself – carried over where blame should go – my wife blaming herself** (G. Emotional Impact-Communication)    - In early 50s, I questioned my health, and was worried what was happening    - “You’re different now (with the gout)”- I had to leave early    - She didn’t know why I was unhappy | **Score: 3** |
| 1. **It hurt and I couldn’t go to my son’s baseball game, Wife would be upset** (B. Social life Interference)    - Son got a sports scholarship, I couldn’t see the game    - Kids says “before dad had this (gout), we did this”    - If your pain makes you suicidal, everything else is secondary    - There were times it hurt so bad; I am divorced now    - Relationship should be on the list of goals    - 2 days before a 20-year reunion, it flared - neither one could go    - Social events    - Causes you to cancel plans- especially a major event      1. Have her do things on her own, like going to football games      2. One of her sorority events – I came down with flare – couldn’t go – she was devastated by it | **Score: 2** |
| 1. **Sleeping arrangement – sometimes with pain you have to sleep separately** (A. Physical Impact-Intimacy)    - Direct effect on intimacy    - Flares can be “deadly”, you can’t move your joints”    - The elimination of intimacy; It just affects your whole life | **Score: 1** |
| 1. **Work related: Gout affected income and us** (F. Treatment-related Financial Burden stressing relationship) | **Score: 0** |
| 1. **Perception that you may have done this to yourself affects your relationships** (A. Emotional Impact-Intimacy)    - Wife says: what did you eat today? Did you drink beer? It’s a question of concern on one hand and on the other hand it feels like she is saying “why did you do that?”    - If she finds out, you’re in a lot of trouble    - My wife never understood that my gout was due to heart disease | **Score: 0** |
| 1. **It affected family due to pain** (B. Social life Interference)    - They didn’t understand    - She knew my heart disease, but didn’t understand my gout | **Score: 0** |
| 1. **It’s still mysterious to me**    - It’s not like going to the dentist | **Score: 0** |
|  | **Total: 18** |
|  |  |
| **NGT #12 12/12/2017 9:00 AM 2Female 2African-American** | |
| 1. **It has affected our finances** (F. Treatment-related Financial Burden stressing relationship)  - It’s stressful for the relationship - Hospital stays are expensive - Medications are expensive even with insurance | **Score: 5** |
| 1. **It has changed our sex lives** (A. Physical Impact-Intimacy)    - Limits your sex life    - When in pain, it’s the last thing on your mind    - When you want to, you can’t because of the pain – frustrates me    - I’ll shoot you right now, if you touch me    - At that point with that pain, can’t see yourself pushing through that pain    - Pain is like being stabbed, punched, and stepped on at the same time    - I have had a fracture and child birth – gout is worse, because it’s a lingering pain, even with the medicine    - Sleeping in different beds is an issue- happens during the flare all the time    - We knew this couple sleeping in different rooms; Laughed and said we’re never going to do that – When I have flare, can’t stand the accidental touch    - Sex is out of the question if in pain | **Score: 3** |
| 1. **It causes distance at times of flare ups** (A. Physical Impact-Intimacy)  - So much goes on with the flare-up with your body that the other person cannot feel – physical distance - When you are in pain – emotional withdrawal – so focused on making the pain stop | **Score: 2** |
| 1. **It has affected the activities we do together** (B. Social life Interference)  - We used to go fishing, take walks in the park – I can’t do that, he went fishing because we went together - We made agreement – date night 1-2 times a month - Haven’t been able to do that due to flare-ups – not being able to have quality time as a couple - Strains our marriage – work on “us” - Flare ups make it hard for us to enjoy regular dates/outings | **Score: 2** |
| 1. **The walking and sitting is an issue and husband does not understand because he doesn’t know the pain or the situation** (C. Trust issues/Understanding)  - Thinks I use gout as an excuse sometimes - A lot of people try to explain your situation, what you say is “boring” - I have had similar situations with doctors - I haven’t been prescribed any medication - Everyone assumes gout affects your toes – I had gout in my wrist - What husband knows about gout is from other family members rather than me; He says, My cousin says if you do this, it will stop - Arguments/misunderstandings about old wives tales - Communicating about what is the issue | **Score: 0** |
| 1. **It has changed the way I cook and he doesn’t understand** (E. Restricted Diet/Food choices negatively impacting the relationship)  - Less fried food, shellfish – don’t want to cook twice - Changed the way I cook – less salt, less fried chicken, pork chops, fish - We have been together for 38 years; he has a certain look he doesn’t have to say it – feeling they have to change with you (and they don’t want to) | **Score: 0** |
|  | **Total: 12** |
|  |  |
| **NGT #13 1/23/2018 9:00 AM 3Male 3African-American** | |
| 1. **Limits activity that requires walking** (B. Disability)  - She likes to go places, thinks that these activities require my presence and my gout does not allow me to do that - She have to drive, if I am having a flare; It’s worse than my kidney stones - I can’t help her with usual household chores during my flare | **Score: 6** |
| 1. **It makes me dependent on my wife during an episode** (B. Physical Dependence)  - She sees me in this pain, there is nothing you can do - Mine is in elbows and knees - Can’t do household chores - I have arthritis and sometimes that affects me too, even when I am not having a flare - Sometimes have to call my son to do things that I would otherwise do | **Score: 5** |
| 1. **My wife would get aggravated, because she did not know what would help me** (G. Emotional Impact-Communication)  - Women are nurturers and she could not resolve how to help me with my pain - She wanted to find a solution for me, just couldn’t - She goes with me to the doctor – She is my snitch to the doctor; we had quite a different view-point about treatments, now we have a shared viewpoint: For years, I declined any medication treatment, I doctored myself- you know you go on the Internet, self-diagnose and treat yourself; it was a difficult obstacle for me to overcome - I always want to look at what doc says, and do my own checking | **Score: 3** |
| 1. **It made her super-conscious of what I could eat, and what to avoid to the extent of limiting her food choices** (E. Restricted Diet/Food choices negatively impacting the relationship)  - We were in Peru and Puerto Rico and went to this Restaurant, super intense shell-fish, I had a gout flare even without eat any shell-fish at all. I guess, may be just being there…since then, my wife and I cut out all beef, all pork, all shell fish from our diets for years; we both became vegans for years, until I got the medication (allopurinol); since being on the medication, I can eat all types of foods. Allopurinol does wonders for me. - Before allopurinol, we eliminated all chicken tenders from our diet, now we can both enjoy it - “Can’t be around shell-fish, you know” - She was aggrevated with the amount of time I was spending to avoid a gout attack – going to the gym, home remedies, things to avoid flares - I was avoiding everything, I though was causing it - The need to take time away from her to constantly go to the Gym | **Score: 2** |
| 1. **Emotional Stress/negative effect on personality: It’s hard enough not to grimace, and complain, I try to be pleasant** (A. Emotional Impact-Intimacy)  - When you are arguing, it’s difficult to be intimate - Emotional vulnerability due to “male ago” - When you are hurting, your wife won’t be aggressive towards intimacy- she will avoid it. - It changes your demeanor, people notice that; of all people, your wife notices that - You appear not to be a happy person and you are not - I hate to deal with people, who complain about their condition; when you have gout, that’s what you start doing - It definitely identified my vulnerabilities to her – Emotional stress on intimacy - Constantly asking, Can you do this? - Every time she would ask me, Is this too far to walk? or how’s the knees?” - Takes a few seconds to get the stance - The activities that we did together- gout had a negative effect | **Score: 2** |
| 1. **If you are having an attack, you are not going to get into “it”** (A. Physical Impact-Intimacy)  - Frequency of intimacy is not there; gout attacks make it impossible - If you have an attack in your toe or foot or knee, forget it | **Score: 0** |
| 1. **It never seemed to interfere with my relationship with my 2 wives** (G. Not in relationship/No or positive effect on relationship)  - Care is so much better, I don’t think it impacted me - That was such a long time ago, I am 82 now - My gout is always in my left foot, I start colcrys the moment I get the 1^st^ sign of a flare | **Score: 0** |
|  | **Total: 18** |
|  |  |
| **NGT #14 2/12/2018 11:00 AM 4Female 4African-American** | |
| 1. **Physical Impact** (B. Disability)  - Gout made me walk with a cane - When I first had it, I couldn’t walk, it hurt so bad; It hurt my leg and feet - Doctor said I had to change my diet - 4-5 years ago, I was in the hospital with my gout - Can’t put my feet on the ground, when it flares - I was diagnosed with gout in 1998, I wouldn’t out my feet on the floor, during a flare - After diagnosis, I was started on a medication, gout always came back, then I stopped shellfish and organ meats, now it’s a little better - I couldn’t do anything at all - Toe swollen, it was diagnosed, I hurt so bad, I couldn’t walk - Husband had to pick me up and put me in the wheel-chair - I am married and “gouch” affected my toe, I couldn’t walk | **Score: 9** |
| 1. **Emotions and Feelings between the two of us (because of the pain)** (A. Emotional Impact-Intimacy)  - The emotional stuff affects the intimacy - My husband wasn’t used to doing all that stuff, helping me physically when I had my first gout attack; even if he was my husband, I was little ashamed - When it occurred, has no warning, hit you unexpectedly, I need some relief | **Score: 7** |
| 1. **Social behavior changed** (B. Social life Interference)  - I am hurting and we have somewhere to go- he will stay at home with me; he is a very outgoing person, now he has to sit home and watch TV - “You can’t do nothing when it hurts that bad” - He would go to the Church and I would stay at home - Grandchildren are jumping on you and you hurt – un..ah, can’t do that - Changed my diet completely after the gout diagnosis; I didn’t drink alcohol before but I used to drink a lot of Pepsi, and I had to stop drinking it | **Score: 4** |
| 1. **I don’t have any relationship right now** (H. Not in relationship/No or positive effect on relationship)  - My gout started after my husband passed away - I don’t have anyone like that since my gout was diagnosed a few years ago; my daughter takes care of me | **Score: 3** |
| 1. **Change in Role/Not communicating with each other** (G. Emotional Impact-Communication)  - Somethings he was taking for granted, all he did was work and pay all the bills; he never cleaned or cooked; with my gout, it gave him some responsibility, and he learnt to do these things, before I died; I was working too, you know. - Now we share role in the household chores - Initially was difficult, but as time went by, it drew us closer | **Score: 1** |
|  | **Total: 24** |
|  |  |
